# Addressing the immediate need for emergency providers in resource-limited settings: the model of a six-month emergency medicine curriculum in Haiti

**DOI:** 10.1186/s12245-018-0182-y

**Published:** 2018-04-06

**Authors:** Shada A. Rouhani, Kerling Israel, Fernet Leandre, Sosthène Pierre, Brennan Bollman, Regan H. Marsh

**Affiliations:** 1 Brigham and Women’s Hospital, 75 Francis Street, Boston, MA 02115 USA; 2 Harvard Medical School, 25 Shattuck Street, Boston, MA 02115 USA; 3 Hôpital Universitaire de Mirebalais, Route Départmentale 11, Mirebalais, Haiti; 4 Partners In Health, 800 Boylston Street, Suite 300, Boston, MA 02199 USA; 5 Zanmi Lasante, 18a Route De Santo, Croix Des Bouquets, Port Au Prince, Haiti; 6 Faculte de Medecine et de Pharmacie de l’Universite d’Etat d’Haiti, 89 Rue Oswald Durand, Port-au-Prince, Haiti; 7 Louisiana State University Health Sciences Center, 433 Bolivar St, New Orleans, LA 70112 USA

**Keywords:** Emergency medicine, Emergency care, Haiti, Education, Training, Emergency providers, Resource-limited

## Abstract

**Background:**

In many resource-limited settings, emergency medicine (EM) is underdeveloped and formal EM training limited. Residencies and fellowships are an ideal long-term solution but cannot meet immediate needs for emergency providers, while short-term programs are often too limited in content. We describe a third method successfully implemented in Haiti: a medium-duration certificate program to meet the immediate need for emergency specialists.

**Methods:**

In conjunction with the Haitian Ministry of Health and National Medical School, we developed and implemented a novel, 6-month EM certificate program to build human resources for health and emergency care capacity. The program consisted of didactic and supervised clinical components, covering core content in EM. Didactics included lectures, simulations, hands-on skill-sessions, and journal clubs. Supervised clinical time reinforced concepts and taught an EM approach to patient care.

**Results:**

Fourteen physicians from around Haiti successfully completed the program; all improved from their pre-test to post-test. At the end of the program and 9-month post-program evaluations, participants rated the program highly, and most felt they used their new knowledge daily. Participants found clinical supervision and simulation particularly useful.

Key components to our program’s success included collaboration with the Ministry of Health and National Medical School, supervised clinical time, and the continual presence of a course director. The program could be improved by a more flexible curriculum and by grouping participants by baseline knowledge levels.

**Conclusion:**

Medium-duration certificate programs offer a viable option for addressing immediate human resource gaps in emergency care, and our program offers a model for implementation in resource-limited settings. Similar options should be considered for other emerging specialties in resource-limited settings.

## Background

There is a tremendous need for improved emergency services in low- and middle-income countries (LMICs) [1–3] to reduce the burden of infectious diseases, non-communicable diseases, and injuries. This burden falls disproportionately on LMICs, where an estimated 90% of trauma-related deaths [4] and 75% of non-communicable disease deaths occur [5]. By one estimate, as much as 45% of global deaths and 35% of global disability-adjusted life years could be addressed through high-quality emergency care [3].

Though the WHO has endorsed emergency care as “an essential part of integrated healthcare delivery,” [6] emergency medicine (EM) remains underdeveloped in these contexts. Educational and training programs are essential to expanding emergency care in LMICs, yet there are few EM-specific medical education programs in these contexts [7]. This educational gap must be addressed to improve clinical outcomes and strengthen health systems.

No single best practice has been established to meet the immediate EM training needs in LMICs. Directly exporting training programs from high-resource settings is not a viable solution, given differences in human resources and health system development. Recently, several LMICs have opened EM residencies and fellowships, ranging in duration from 1 to 6 years [8–13]. Over the long term, these programs create a cadre of highly trained emergency physicians (EPs). Yet, the duration of training and limited number of trainees mean these programs cannot fill the urgent human resource gap. For example, a three-year residency program with five residents per cohort would take 13 years to train just 50 providers.

An alternate training approach is short courses of several days to weeks [14, 15], often conducted by outside organizations, sometimes in collaboration with state and non-state actors. By design, these condensed trainings focus on specific high-yield topics rather than the broader clinical reasoning required in EM and do not provide a pathway to certify providers to fill long-term health system needs. Participants may improve their “book knowledge” but often struggle to apply new information to patient care. Further, since these trainings are not longitudinal, there is no chance to refine skills and knowledge over time.

We propose a novel third method: a medium-duration certificate to meet the immediate need for emergency specialists. We describe the implementation of such a certificate program in Haiti, a low-income country with representative human resource and health system gaps in emergency care.

## Methods

### Medical education in Haiti

There are five medical schools in Haiti, who together graduate approximately 350 students annually. Each has a 6 or 7-year curriculum with the final year devoted entirely to clinical rotations. In addition, the health system welcomes Haitians who have graduated from medical schools abroad, including from Cuba through the Haitian-Cuban cooperation. There is no formal EM training at either the medical or nursing schools throughout the country.

To become licensed, medical school graduates complete a mandatory social service year, in which they are assigned to work at hospitals around the country by the ministry of health. Though some placements are urban with more senior physicians, many placements are rural and new graduates may be the only physician at the facility. New graduates are not formally supervised during this social service year; after completion of social service, they become licensed as generalist physicians.

Licensed generalist physicians can apply for a residency program, but less than a third of the applicants get a residency position (Morse M, Pierre P. HUM Medical Education Prospectus. Unpublished). Although Haiti has increased its graduate medical education capacity since the 2010 earthquake, residency opportunities remain limited while the number of applicants increases. Physicians who do not apply for or receive a residency acceptance continue to work as generalist physicians. Generalists work in both the public and private sector at primary and secondary levels of health care.

### Local setting

Haiti has few post-graduate medical training programs and, prior to October 2014, had no EM residency program or emergency physicians [16]. EM services in Haiti are limited and, when available, are often delivered by generalist or social service physicians with no emergency care training. In 2013, Partners In Health and the Haitian government opened Hôpital Universitaire de Mirebalais (HUM), a 300-bed academic referral hospital with a 21-bed emergency department (ED), to address the country’s clinical and medical education needs. The HUM ED sees all non-obstetric emergencies and has dedicated 24/7 nurse and physician staffing, a rarity for EDs in Haiti. At the time of the certificate program, there was no formal EM training or EM specialists in Haiti.

In response to requests from and in collaboration with the Ministry of Health and National Medical School, we created a 6-month Certificate in EM to train physicians in the core competencies of the specialty and address a national training gap.

### Program participants

Fourteen physicians participated in the certificate program, including all eight HUM ED physicians and six physicians with leadership roles in other Haitian EDs (from five public hospitals and one non-governmental organization hospital). All three other academic hospitals in Haiti sent participants, as did two nearby district hospitals and a non-governmental organization trauma hospital in Port-au-Prince. Hospitals nominated their representative individual participants, chosen for their leadership skills and likelihood to teach others. Clinical supervision needs dictated the maximum number of participants. Four participants had prior residency training (three family medicine, one internal medicine), and the remainder were generalist physicians. None of the participants had prior emergency medicine training. Though all participants were currently working in EDs, some (including all eight HUM ED physicians) had worked in EDs for less than 1 year. The certificate program was free of charge to all participants, who remained on full salary at their home institutions during the program.

### Program components

The program consisted of didactic lectures, simulation, and clinical supervision. The didactic component was held every other week, allowing participants to work full-time at their home hospitals and reduce transportation costs. To graduate, participants were required to attend 75% of didactic lectures, complete 180 supervised clinical hours, take a pre- and post-test, prepare and give one lecture, and submit a case and procedure log. The pre- and post-tests were written by a panel of emergency physicians (EPs) with global health expertise and experience in Haiti. Questions were peer-reviewed by the course directors.

### Didactic curriculum

The 96-h didactic program focused on core EM competencies and included lectures (76 h; Table 1) supplemented by a variety of other teaching modalities. Simulation consolidated critical care knowledge and taught a team-based approach to patient management. Hands-on skill-sessions reinforced procedural competencies such as cardiopulmonary resuscitation, noninvasive ventilation, ultrasound, and splinting. Journal clubs introduced the review of medical literature, and morbidity and mortality conferences modeled case review for quality improvement. As part of the lecture curriculum, all participants gave a 1-h presentation on an assigned key content area with mentorship from program directors. The curriculum prioritized emergency topics with a high local burden of disease and potential for morbidity and mortality if incorrectly treated. Didactics included the gold standard for diagnosis and treatment, but focused on practical approaches adapted to the local context. Some common EM topics, most notably intubation and ventilation management, were not taught given the paucity of ventilators available in the country at the time of the program.

**Table 1:** Didactic curriculum: subject areas and representative lecture topics (including participant lectures)

Content area	Hours	Example lecture(s)
Burn, trauma, and orthopedics	14	• Orthopedic emergencies by extremity • Non-accidental trauma • Head and spinal trauma • Abdominal trauma and hemorrhagic shock
Cardiology	10	• Congestive heart failure and cardiogenic shock • Dangerous chest pain: myocardial infarction, pulmonary embolism, and aortic dissection • ECG interpretation
Infectious diseases	10	• Intracranial infections • Tropical diseases: malaria, dengue, and chikungunya
Gastroenterology	6	• Diarrheal illness, dehydration, and fluid resuscitation • GI and intraabdominal surgical emergencies
Respiratory	6	• Approach to dyspnea • Management of acute asthma and COPD
Neurology and psychiatry	5	• Status epilepticus and seizure • Altered mental status and coma • Acute psychiatric emergencies
Advanced life support^a^	4	• Advanced Cardiac Life Support principles (ACLS) • Pediatric Advanced Life Support principles • Neonatal resuscitation
Toxicology and Environmental health	4	• Environmental emergencies: lightning, drowning, and electric shock
Imaging^b^	3	• Ultrasound in shock
Endocrine	2	• Diabetic ketoacidosis and hyperosmolar syndrome
Hematology	2	• Sickle cell emergencies
Obstetrics and gynecology^c^	2	• Vaginal bleeding in pregnant and non-pregnant patients
Renal	2	• Acute renal failure and hyperkalemia
Other pediatric topics^d^	2	• Noninfectious neonatal emergencies
Principles of EM	1	• Approach to a patient in the ED
Dermatology	1	• Life-threatening rashes
Genitourinary	1	• GU emergencies: the urinary tract and penile pathology
Ophthalmology	1	• Ophthalmologic emergencies

^a^ In addition to the 4 h of advanced life support topics, 12 h of the topics classified by organ system above were on critical care topics, for a total of 16 h of critical care lectures

^b^ One hour of ultrasound training was optional for participants without any prior exposure to ultrasound

^c^ In addition, a lecture on trauma in pregnancy is classified under burn, trauma, and orthopedics

^d^ An additional 5 h of pediatric topics are classified by organ system, for a total of 7 h on pediatric specific topics

### Clinical supervision

In the absence of Haitian emergency physicians (EPs), international EPs provided clinical supervision: two US-trained EPs together provided full-time program leadership and supervision, and eight visiting faculty each volunteered for 3–4 weeks. On average, two supervising emergency physician (EP) faculty were at HUM at any time throughout the program. Participants completed at least 180 h of supervised clinical time at HUM. Supervision schedules were designed to minimize participant travel, assure oversight, and avoid ED overstaffing.

Participants maintained a case log, documenting both 100 supervised cases and pre-specified numbers of essential procedures, such as adult and pediatric resuscitations, thoracentesis, and splinting/casting (Table 2). As an essential component of EM, bedside ultrasound was prioritized and participants were required to perform at least ten each of FAST, cardiac, and lung ultrasounds.

**Table 2:** Minimum number of required cases and procedures for certificate program participants

Supervised cases	100
Supervised procedures	
Adult resuscitation	10
Pediatric resuscitation	5
Trauma resuscitation	5
Management of respiratory distress	10
Splinting or casting	10
Dislocation reduction	3
FAST exam (ultrasound)	10
Lung ultrasound	10
Cardiac ultrasound	10
Paracentesis	3
Thoracentesis	3

### Program costs

To minimize costs, we relied on volunteer visiting faculty for whom we subsidized only in-country expenses, although this resulted in frequent turnover of clinical supervisors. The co-course directors were also the department co-chairs and were not hired specifically for this course. Participants were not compensated; participants not from HUM received local food and housing but not transport. Clinical time in the HUM ED did not add additional operating costs.

## Results

**Fig. 1:**
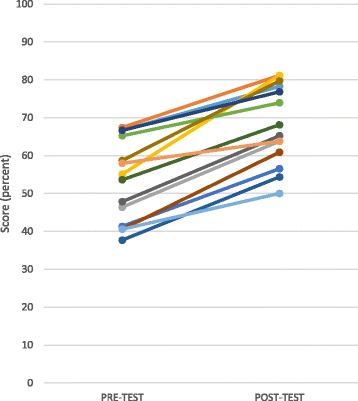
Participant pre-test and post-test scores. Each line represents the pre- and post-test scores of an individual participant. All participants showed improvement between the pre- and post-test

All 14 participants completed the certificate program. One year later, 11 were still working in EDs. All participants improved on their 56 question pre-/post-test with an average improvement of 15 percentage points (Fig. 1).

Upon completion of the program, all participants filled out a program evaluation with an average overall rating of 4.3 out of 5. Highly rated program components included simulation, clinical supervision and the participant presentations. The content areas identified as the most valuable were advanced life support and resuscitation, imaging (ultrasound and CT), cardiovascular emergencies and ECG interpretation, pediatric emergencies, dyspnea, and the philosophy of emergency medicine (including how to approach an emergency patient, how to simultaneously diagnose and treat a patient, and how to move patients through an emergency department).

Participants were asked to anticipate barriers to applying their new knowledge. One common barrier was resource limitations at their home institutions compared to HUM. Others included patient education, late-stage presentations, motivating other staff, and the perceived difficulty in changing culture at their home institutions.

Nine months after the program, a second evaluation was sent to the participants. Nine (64%) responded, all of whom still worked in EDs. Most reported using knowledge from the certificate program on a daily basis (daily: 67%, 2–4 times/week: 22%, 1–2 times/week: 11%). When asked to rate the importance of their supervised clinical time on their education, eight (89%) felt it was “very important.” Program components valued at 9 months included the supervised clinical experience, simulations and structured debriefs, and specific clinical topics including orthopedic emergencies, shock, trauma management, and ECG interpretation.

## Discussion

This medium duration, 6-month certificate program in Haiti, represents a novel approach to address gaps in human resources for health and can be replicated for emergency care and other new specialties in similar settings. Notably, the program improved participants’ EM knowledge and was well received by participants, the Haitian government, and the national medical school.

### Critical components for success

Several structural components were critical to the program’s success. First, the engagement of the Haitian Ministry of Health and National Medical School allowed official certification of the program and facilitated participation of physicians from multiple public hospitals. Second, training physicians from throughout the country disseminated knowledge, enhanced recognition of EM as a specialty, and fostered an EM professional network.

Third, we recruited both international EPs and national non-EP specialists as program faculty. Visiting EPs were crucial for teaching core EM content and role-modeling the clinical approach to the emergency patient. For select topics, national specialists from other disciplines taught didactic content related to their expertise, with explicit guidance on learning objectives important for EPs. For example, a Haitian obstetrician-gynecologist lectured on first trimester obstetric emergencies. This involvement in the program increased recognition of EM among other specialties, and their lectures were inherently adapted to local context.

Fourth, the continuous presence and leadership of a course director was essential, facilitating coordination and real-time management of contingencies, as well as orienting international EPs to the local standards of care, knowledge level of participants, and successful teaching strategies. This allowed successive visiting faculty to maintain consistent focuses on the strengths and weaknesses of individual participants and difficult content areas. Course directors provided consistency for participants, ensuring that requirements were met and adapting the curriculum as needed.

Finally, clinical supervision and simulation were essential to the program’s success. Direct clinical supervision helped participants learn the approach to the emergency patient and apply knowledge from didactics. Topics covered briefly during didactics, such as image interpretation, were explored in depth at the bedside. Importantly, case logs facilitated clinical supervision, gave objective performance measures, and structured the relationship between participants and visiting faculty. In addition, simulation taught simultaneous stabilization, diagnosis, and treatment with a focus on low-frequency, but high-risk emergencies. Simulation also offered a safe environment for students to learn without posing risk to patients.

### Areas for improvement

The program could be improved by structuring more flexibility into the curriculum to accommodate events from illnesses to unforeseen opportunities for specialist lectures. Further, some didactic areas (e.g., ECG interpretation) required more time than anticipated. Similarly, due to clinical variability during ED shifts, not all participants met the pre-specified procedure targets, and we offered flexibility in this requirement.

Future programs could group participants by baseline knowledge level; the varied baseline knowledge level among our participants necessitated adapting lectures to ensure content was appropriate for all learners. Lastly, translation provided an unexpected challenge, as the hospital English-French translators lacked sufficient medical knowledge to interpret nuanced clinical concepts. In the end, our participants provided real-time translation themselves (by those fluent in English) to maximize group education.

Regarding sustainability, our conclusion at the end of the program was that the 6-month certificate alone is not sufficient as a train-the-trainer model. While students improved their clinical EM skills—the primary objective of the course—they did not reach a level sufficient to become teachers themselves. Future iterations should consider alternate ways to develop local trainers, either by repeating the course multiple times, providing additional clinical supervision and accompaniment before or after the course, and/or pairing the certificate program with residency training, so that local residency-trained EPs could ultimately run the certificate course.

### Future directions

Medium-duration certificate programs offer a novel approach to address the need for qualified emergency providers in LMICs by offering greater breadth and depth than traditional short courses while expeditiously training more providers than a residency: in the same 13 years needed to graduate 50 residency-trained EPs, an annual certificate program could train 180 providers. In most of Haiti, like many LMICs, generalist physicians with limited training provide front-line emergency care. Even though Haiti now has an EM residency program, placing specialists in remote hospitals will not be realistic anytime in the foreseeable future. Certificate-trained generalist providers could help bridge these human resource gaps. Further, this program could help link remote generalist providers to teaching hospitals where the training is run to create long-term educational support and transfer networks. For these reasons, the Haitian Ministry of Health and National Medical School have requested that the emergency certificate course be offered on an ongoing basis.

Some questions remain unanswered. Our program was designed to meet Haiti’s needs. We anticipate the content is generalizable to other LMICs, but this warrants further evaluation, as does knowledge retention after the course. Additionally, the role of a certificate program in a country with a new EM residency is undetermined. The coexistence of certificate-trained and residency-trained physicians could impact the new specialty as it seeks professionalization. Finally, this program was successful for generalist and specialist physicians with no prior EM training, over half of whom had worked in EDs for less than a year. Thus, we anticipate that this program is relevant to all physicians, particularly recent graduates and those with limited prior EM experience. The adaptability of this curriculum to other cadres of providers, such as nurses or midlevel providers, remains to be seen. However, the program’s design, covering core EM content, may allow easy modification for medical students, residents in other specialties, and/or midlevel providers to facilitate task-shifting.

## Conclusions

The implementation experience and lessons learned from this 6-month curriculum in Haiti offer a starting point for future programs. Given the urgent need for qualified emergency providers in LMICs, medium-duration EM certificate programs can play a critical role in addressing the immediate human resources gap to provide high-quality emergency care. Similar human resource gaps for other medical specialties also exist in LMICs, and medium-term certificate trainings warrant exploration in these fields as well.

## Abbreviations

ED: Emergency department
EM: Emergency medicine
EP: Emergency physician
HUM: Hôpital Universitaire de Mirebalais
LMICs: Low- and middle-income countries
